# Economic Evaluation of Long-Term Survivorship Care for Cancer Patients in OECD Countries: A Systematic Review for Decision-Makers

**DOI:** 10.3390/ijerph182111558

**Published:** 2021-11-03

**Authors:** David Brain, Amarzaya Jadambaa

**Affiliations:** Australian Centre for Health Services Innovation, Centre for Healthcare Transformation, School of Public Health and Social Work, Queensland University of Technology, 60 Musk Avenue, Brisbane, QLD 4059, Australia; a.jadambaa@qut.edu.au

**Keywords:** cancer, long-term survivorship, economic evaluation, health economics, systematic review, decision-making, health services

## Abstract

Long-term cancer survivorship care is a crucial component of an efficient healthcare system. For numerous reasons, there has been an increase in the number of cancer survivors; therefore, healthcare decision-makers are tasked with balancing a finite budget with a strong demand for services. Decision-makers require clear and pragmatic interpretation of results to inform resource allocation decisions. For these reasons, the impact and importance of economic evidence are increasing. The aim of the current study was to conduct a systematic review of economic evaluations of long-term cancer survivorship care in Organization for Economic Co-operation and Development (OECD) member countries and to assess the usefulness of economic evidence for decision-makers. A systematic review of electronic databases, including MEDLINE, PubMed, PsycINFO and others, was conducted. The reporting quality of the included studies was appraised using the Consolidated Health Economic Evaluation Reporting Standards (CHEERS) checklist. Each included study’s usefulness for decision-makers was assessed using an adapted version of a previously published approach. Overall, 3597 studies were screened, and of the 235 studies assessed for eligibility, 34 satisfied the pre-determined inclusion criteria. We found that the majority of the included studies had limited value for informing healthcare decision-making and conclude that this represents an ongoing issue in the field. We recommend that authors explicitly include a policy statement as part of their presentation of results.

## 1. Introduction

Cancers of all types are a global health concern, and the worldwide impact of cancer is expected to continue to increase in the coming decades [1]. Long-term survivorship care for cancer patients is a crucial component of a well-functioning healthcare system, but the ongoing management of survivors comes at a cost. Improved treatment has accelerated progress against cancer and has driven a record drop in overall cancer mortality, leaving healthcare decision-makers to face multiple challenges [2,3,4,5]. Firstly, the current healthcare environment is characterized by finite budgets and high expectations of good health outcomes, where healthcare decision-makers are required to balance non-increasing budgets with an increased demand for services [6]. This is a challenge for decision-makers who find themselves with an increased number of cancer survivors who require ongoing, long-term support services. Secondly, healthcare decision-makers are required to quickly synthesize evidence from a range of competing disciplines regarding service provision, so the ease with which findings can be translated into practice, or at the very least pragmatically interpreted, is of significant importance to them [7]. Despite increased interest and reliance on economic evidence in healthcare, decision-makers need to understand the potential impact of acting on such evidence and how such actions might influence clinical outcomes and costs. Healthcare decision-makers require economic evidence to be high-quality, useful for informing resource allocation decisions, applicable to the real-world healthcare setting and easily translated into practice [8,9].

Systematic reviews of economic evaluations relating to long-term cancer survivorship exist, but they do not focus on the usefulness of reported evidence for decision-making. One review focuses on economic evaluations of follow-up cancer care treatment [9], while another was conducted to identify analyses that have been included in guidance on cancer follow-up by UK government agencies and aimed to assess the relevance to the UK setting [10]. The most recent review (2021) focused only on physical activity interventions for cancer survivors in developed countries [10]. The aim of our study was to conduct a systematic review of the available economic evaluations of long-term survivorship care for cancer patients in OECD countries. Our intention is to support healthcare decision-makers—clinicians, policymakers and budget allocators—by summarizing the best available evidence associated with the provision of long-term cancer survivorship care and assess included studies for both quality and usefulness from a health economics perspective. To our knowledge, this work has not been undertaken elsewhere, and our results add novel information to the evidence base.

## 2. Materials and Methods

This study followed the PRISMA statement [11] for processing and reporting systematic reviews (Table A1 and Table A2, Appendix A). The aim of this process was to capture all relevant economic evaluations of long-term survivorship for cancer patients in OECD countries. A review protocol was developed in advance, with search methods and inclusion criteria specified (Table A3, Appendix B).

### 2.1. Inclusion and Exclusion Criteria

This systematic review included studies meeting the following inclusion criteria: (1) reported original empirical research published in a peer-reviewed journal; (2) evaluated the economic impact and health outcomes associated with implementing long-term survivorship care for cancer patients who had initial cancer treatment(s)—any economic evaluation. This may include either model-based or non-model-based economic evaluations such as cost–utility analysis, cost-effectiveness analysis and costing analysis; and (3) the study was conducted in OECD countries. The most widely used definition of cancer survivorship is from the National Coalition for Cancer Survivorship and includes for each person the period ‘from the time of diagnosis, through the balance of his or her life, regardless of the ultimate cause of death’ [12]. Different stages of survivorship comprise acute (diagnosis to treatment), chronic (ongoing) and long-term/late survivorship (≥5 years post-diagnosis) [13]. The target population of this review is cancer patients of any age and gender who have received survivorship care for ≥5 years after initial cancer treatment. As we aimed to include economic evaluations from the payer’s perspective, costs related to follow-up care (e.g., direct and indirect medical costs, intervention costs and overhead costs) and any outcome (e.g., recurrence, detected relapse, quality-adjusted life years (QALYs) and life years (LYs)) are reviewed in this review. Studies were excluded if they evaluated follow-up care for hyperplasia/dysplasia or management of chemo/radiotherapy-induced symptoms. Scholarly reviews, letters to the editor, comments, news and conference abstracts were also excluded. In the few instances where the same data were reported across different publications, the most informative article was selected: for example, a study reporting the full set of cost-effectiveness results from a model comparing alternative follow-up schedules for women across four different risk profiles was selected [14] ahead of one providing results that only take into account age and adherence to mammography [15]. Final decisions regarding the inclusion or exclusion of studies were made based on a consensus between both reviewers (A.J. and D.B.). The full inclusion and exclusion criteria used for selection of the studies included in this review are shown in the protocol published in PROSPERO (ID: CRD42020218966) as well as in Appendix B. 

### 2.2. Search Strategy

Five electronic databases, namely MEDLINE, PubMed, PsycINFO, National Health Service Economic Evaluation Databases and Health Technology Assessment Databases, were searched to identify studies conducting an economic evaluation of long-term survivorship care for cancer survivors in OECD countries. The following search terms were used: “economic evaluation*” or “economic analys*” or “cost*” and “follow-up” or “survivorship care” or “long-term strateg*” and cancer* or carcinoma* or neoplasm*. The search was restricted to the English language and by publication period between 1 January 2000 and 12 November 2020. The reference lists of included studies were searched for other relevant studies.

### 2.3. Data Extraction and Quality Assessment

After preliminary screening of the title and abstract, articles deemed relevant were retrieved for examination. Data extraction sheets were pilot tested and revised to include the data source, study design, period of publication, location, sample size, age group, type of cancer, intervention/comparator, type of economic evaluation, presence of sensitivity analysis and main results (Table A5, Appendix C). 

The quality of each study was assessed using the Consolidated Health Economic Evaluation Reporting Standards (CHEERS) statement [16]. The 24-item checklist is a consolidation and update of previous reporting guidelines and consists of recommendations on reporting methods and findings for economic evaluations (Appendix D). It also provides an example to ensure more consistency and transparency when reporting results and can be used as a way of comparing studies. Each item in the checklist was scored as having either met the criteria in full (1), partially (0.5) or not at all (0) or as not being applicable (NA). Overall compliance with the checklist was assessed by calculating the proportion of the CHEERS criteria addressed by the study. Fully meeting the criteria would contribute 1 to the numerator while partially meeting the criteria would contribute 0.5 to the numerator. Any criteria that were not applicable to the study were excluded from the denominator. The quality assessment for each study is presented in Appendix D. While examining the analysis type and findings according to the CHEERS checklist is performed to assess the quality of reporting [17], checking the usefulness to decision-makers is arguably of greater importance [9]. Consequently, we used an adapted version of the approach that has been used in previous systematic reviews to assess a paper’s usefulness to decision-makers [8,9]. This approach assesses usefulness for decision-makers based on the reporting of effectiveness and cost outcomes and the uncertainty associated with such outcomes. We also searched for a clear statement regarding policy implications or directions that should be followed as a result of the study’s outcomes, culminating in an overall usefulness rating of “limited”, “moderate” or “strong”. These ratings can be seen in Table for Usefulness of reviewed studies to decision-making.

## 3. Results

**Figure 1:**
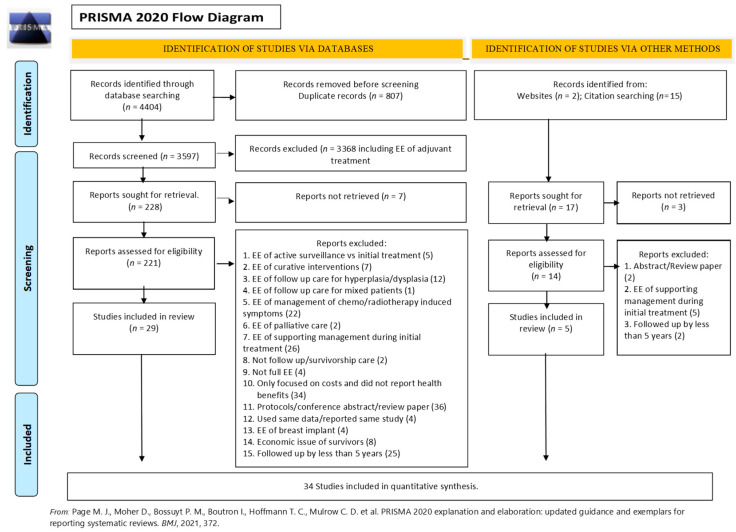
PRISMA flow diagram showing the process of study selection for inclusion in the systematic review.

A total of 4404 articles were identified in the electronic database search, of which 807 were duplicates. The titles and abstracts of 3597 unduplicated references were reviewed and a further 3369 articles were excluded. Seventeen records were identified from additional sources. Reports were not retrieved for 10 studies. Of the 235 studies assessed for eligibility, 34 satisfied the pre-determined inclusion criteria (Figure 1). 

### 3.1. Overview of Included Studies 

Table 1 provides an overview of the descriptive information of the 34 included studies. Studies from the UK and USA were the most common, and almost half the studies retrospectively analyzed cost and effectiveness data. The majority of the studies evaluated survivorship care for colorectal and breast cancer survivors. Twenty out of the 34 included studies were published before 2013, which is when the CHEERS checklist became available [16]. 

The study characteristics of all included studies are summarized in Appendix C. Most studies (*n* = 12) were cost-effectiveness analyses (CEAs), followed by costing-only studies and cost–consequence analyses (CCAs) (*n* = 11), cost–utility analyses (CUAs, *n* = 9) and cost minimization analyses (CMAs, *n* = 2). The outcome measures used in these studies varied according to the study type and design. Ten of the 18 studies that used decision analytic models reported outcomes using incremental cost-effectiveness ratios (ICERs)—calculated by dividing the difference in cost between two alternatives for survivorship care by the difference in their effectiveness. Other studies reported cost per QALY gained, cost per cancer recurrence or change in costs and outcomes separately rather than in a ratio. 

### 3.2. Studies of Long-Term Survivorship Care by Cancer Type

For descriptive purposes, the studies were divided into eight groups depending on the disease condition of interest. These eight groups were colorectal cancer, breast cancer, skin cancer, cervical cancer, head and neck cancer, Hodgkin’s disease, testicular cancer and other cancers. 

#### 3.2.1. Colorectal Cancer

Seven studies assessed the cost-effectiveness of long-term survivorship care in patients previously treated for colorectal cancer. Three were retrospective data analyses [18,19,20] and the remaining four were model-based [21,22,23,24]. Staib et al. [20] estimated the cost per recurrence detected through the existing intensive follow-up strategy in the German setting, which was estimated to be EUR 6000 from a hospital perspective. Bleeker et al. [18] compared the value and effectiveness of different diagnostic tools used to identify potentially treatable recurrences among Dutch patients. They concluded that carcinoembryonic antigen testing (CeA), chest radiography and routine physician visits appear less cost-effective than ultrasonography, computed tomography (CT) and colonoscopy, which can identify most recurrences at a lower health system cost. However, no sensitivity analysis was conducted to test the robustness of the outcomes of these two studies. Borie et al. [21] built a Markov model to compare standard and simplified follow-up examinations for patients after curative colorectal cancer resection in France and found that the ICER for standard versus simplified follow-up would be EUR 3114, substantially lower than the current threshold of acceptability in France (EUR 105,656/QALY) [25]. Renehan et al. [24] developed a model to compare an intensive follow-up strategy with a conventional strategy for colorectal cancer survivors of 5 years or more from the UK NHS perspective. They found that the cost per life year gained was GBP 3402—substantially lower than the NHS threshold for cost-effectiveness, which is GBP 30,000. In another UK study, Macafee et al. [22] used retrospective data for a five-year projection comparing an intensive follow-up strategy with a standard follow-up strategy, concluding that an intensive follow-up would cost an additional GBP 15.4 million over 5 years, with a cost per additional resectable recurrence of GBP 18,077. An Italian study compared several combinations of diagnostic tests for follow-up of patients after curative resection of colorectal cancer [19]. The combination of physical examination, rigid sigmoidoscopy, thorax–abdominal CT and CeA testing was found to be the most cost-effective strategy to monitor stage III and IV colorectal cancer, while physical examination, colonoscopy, thorax–abdominal CT and CeA testing were found to be the most cost-effective methods to monitor stages I and II of colon cancer. Finally, in a more recent UK study, Mant et al. [23] conducted a randomized control trial (RCT) and built a pre-trial economic model to compare different follow-up strategies from the UK NHS perspective. They found that the incremental cost per patient, compared with the less intensive care, ranged from GBP 40,131 with CeA testing to GBP 43,392 with hospital-based imaging to GBP 85,151 with CeA testing and CT combined.

#### 3.2.2. Breast Cancer

Six studies assessed the cost-effectiveness of long-term survivorship care in patients previously treated for breast cancer: one was an RCT, two assessed retrospective audit data and the remaining two were model-based studies. In an Australian study, Grogan et al. [26] retrospectively assessed the costs and effectiveness of several follow-up schedules for women diagnosed with stage I or II breast cancer. They found that three-monthly visits for 4 years and yearly visits in the fifth year cost AUD 1097 per woman. This was a more cost-effective option compared to monthly visits for 5 years, which was more expensive at AUD 3870 per patient. Kokko et al. [27] conducted an RCT to compare four follow-up schedules which differed in visit frequency and in the intensity of diagnostic examination. The total cost of follow-up per recurrence was EUR 4983 lower in the least intensive strategy than in the most intensive follow-up strategy. This amount, EUR 4983 per recurrence, could be saved if visits were only every sixth months and diagnostic tests were taken only when clinically indicated compared to quarterly visits and routine diagnostic tests. Robertson et al. [28] built a Markov model, finding that the most cost-effective strategy in the UK setting was surveillance with mammography alone, provided every 12 months. The incremental cost-effectiveness ratio (ICER) for this strategy compared to no surveillance was GBP 4727 per QALY gained. Lu et al. [29] built a simulation model in the Netherlands to compare the cost-effectiveness of the current guideline-based follow-up with three less intensive follow-up strategies. They found that the current guideline-based strategy was the most expensive and the less intensive programs did not decrease the detection rate of small tumors. They concluded that a reduction in hospital follow-up time by shifting to the National Screening Program or the use of general practitioners and the exclusion of physical examination after 2 years of follow-up was the most cost-effective option, with an estimated cost of EUR 62,100 to increase the detection of small tumors by 1%. However, a sensitivity analysis was not conducted to test the robustness of the outcome of these three strategies. An Australian study used a discrete event simulation model to analyze three alternative mammographic follow-up schedules for postmenopausal women who had treatment for primary breast cancer [14]. After conducting a probabilistic sensitivity analysis, the authors concluded that for most postmenopausal women, annual mammographic follow-up may not be cost-effective, and for women with excellent tumor prognosis, two-yearly follow-up mammograms are most likely to be cost-effective, regardless of age.

#### 3.2.3. Skin Cancer

Three studies assessed the cost-effectiveness of long-term survivorship care in patients previously treated for cutaneous melanoma—one of which retrospectively analyzed data, while the remaining two were model-based studies. Hengge et al. [30] built a Markov model for locoregional recurrence and metastatic recurrence and compared the current intense follow-up strategy with a revised or reduced guideline. The authors found that savings for the 5-year program would total EUR 506,280, and the cost for staging per QALY accounted for EUR 63,252 for the more intense schedule as opposed to EUR 42,433 for the revised, new schedule. The primary outcome of this study was presented as cost per QALY, which enabled direct comparison with other studies. Leiter et al. [31] analyzed retrospective audit data and reported that physical examination was the most effective method, detecting 50% of recurrences, and gave patients a better quality of life. From the perspective of the payer, a risk-adapted surveillance strategy for stages I to II—including thorough history, physical examination and lymph node sonography but omitting CR, blood work and abdomen sonography—seems appropriate and cost-effective. The cost-effectiveness of different radiological examinations was assessed by Podlipnik et al. [32]. Podlipnik et al. [32] built a decision tree model programmed to model a 5-year period and reported that CT scan was cost-effective in the first 4 years (cost-effectiveness ratio ranged between EUR 4710 and 14,437/patient with metastasis) and brain MRI was cost-effective during the first year (cost-effectiveness ratio of EUR 14,090/patient with metastasis). These results were supported by one-way sensitivity analysis.

#### 3.2.4. Cervical Cancer

Three studies assessed the cost-effectiveness of long-term survivorship care in patients previously treated for cervical cancer—two assessed retrospective audit data and one was model-based. An Italian study [33] retrospectively analyzed data on a simplified follow-up diagnostic approach as well as a standard follow-up procedure and reported that a simplified diagnostic approach, which included squamous cell carcinoma (SCC) assay and gynecologic examination, can detect a high rate of recurrence, with a favorable cost-effectiveness outcome. The remaining two studies were conducted in the UK. Baena-Cañada et al. [34] assessed the costs, health-related quality of life and patient satisfaction results of follow-up strategies in primary care compared with specialist-led care, reporting that the costs of follow-up in primary care were lower than those in specialist-led care, with no difference recorded in health-related quality of life (HRQoL). No sensitivity analysis was conducted. As a model-based economic evaluation, Auguste et al. [35] used effectiveness data from a systematic review [36] supplemented with data from other sources to run a model over 5 years. With PSA, the researchers concluded that the use of positron emission tomography/computed tomography (PET/CT) in the diagnosis of recurrent or persistent cervical cancer in a secondary care setting is not cost-effective from the NHS perspective.

#### 3.2.5. Head and Neck Cancer

Two studies assessed the cost-effectiveness of long-term survivorship care in patients previously treated for head and neck cancer. Shah et al. [37] conducted a retrospective cohort analysis comparing standard follow-up—which consisted of routine clinical follow-up every 3 months for 2 years, every four months in the third year and every six months in the fourth and fifth years—with reduced follow-up—which consisted of routine clinical follow-up every six months. They found that the hospital cost savings per patient from reduced review were AUD 5012 over five years, while there was no difference in the time to detection of recurrence or proportion of radically treatable recurrences. Meregaglia et al. [38] provided strong evidence on the cost-effectiveness of the use of intensive radiological assessment in routine surveillance after treatment for head and neck cancer compared to a more minimal option—symptom-driven surveillance. They reported that routine surveillance with the intensive program would be cost-effective, which was supported by two-way sensitivity analysis. More than two-thirds of the Monte Carlo simulations were below the willingness-to-pay threshold of EUR 40,000, indicating that the intervention was cost-effective.

#### 3.2.6. Hodgkin’s Disease

Two economic evaluations of long-term survivorship care strategies for patients previously treated for Hodgkin’s disease were found. A retrospective review of patients treated for Hodgkin’s disease in Canada was performed to evaluate the utility of the components of a follow-up strategy [39]. Dryver et al. [39] concluded that most true relapses are clinically symptomatic, and routine CT is an expensive and inefficient mode of routine follow-up. Supporting these findings were the results from an American study that found that the use of CT in routine follow-up for patients diagnosed at any stage of disease was less effective and more costly than non-CT modalities [40].

#### 3.2.7. Testicular Cancer

Two economic evaluations of long-term survivorship care strategies for patients previously treated for testicular cancer were found. Clasen et al. [41] analyzed the value of routine post-treatment follow-up strategies for patients with seminoma after radiotherapy and reported that abdominal sonography had the highest cost-efficiency among all technical follow-up investigations in the German setting. Charytonowicz et al. [42] built a Markov model to simulate the impact of the miRNA test on testicular germ cell tumor (TGCT) aftercare costs and found that applying this model to the US healthcare system by replacing CT scans with the miRNA test has the potential to save up to USD 69 million per year in aftercare expenses related to TGCT treatment, with exact savings depending on the adoption rate and test price.

#### 3.2.8. Others

Nine additional records on long-term survivorship care strategies for other cancer types were found—five assessed retrospective audit data and four studies used a decision analytic model. In a Canadian study, Gilbert et al. [43] assessed the costs and effectiveness of follow-up surveillance after limited-stage non-small cell lung cancer resection and found that the cost per recurrence detected by a thoracic surgeon is higher than that from using a family physician. The costs of two surveillance strategies in patients after radical nephrectomy for localized primary renal cell carcinoma (RCC) were evaluated in a Canadian retrospective cohort study [44]. Dion et al. [44] concluded that the new Canadian Urological Association surveillance strategy in RCC follow-up was appropriate and cost-effective in comparison with older follow-up strategies. In an American study, Rettenmaier et al. [45] reviewed the surveillance of uterine cancers and found that the CA-125 assay appeared to be the most cost-effective method in following patients with epithelial uterine malignancies compared to serial imaging, vaginal cytology and imaging in the follow-up of uterine cancer. Additionally, the CA-125 assay appeared to be the most cost-effective method in following patients with ovarian cancer and/or primary peritoneal cancer (PPC) compared to CT imaging, vaginal cytology and imaging in the long-term follow-up strategy [46]. Imran et al. [47] compared outcomes and costs for low-risk thyroid cancer patients followed by multidisciplinary clinics in tertiary clinics versus those discharged at 24 months for follow-up in the primary care setting in Canada and reported that the rates of recurrence were similar in both groups, while both healthcare costs and travel costs related to primary care were lower than those in tertiary care. The researchers of these five studies conducted retrospective data analyses without a sensitivity analysis. 

Dansk et al. [48] built a mixed model to assess the economic impact of using hexaminolevulinate hydrochloride-guided blue-light flexible cystoscopy (HAL BLFC) compared with using white-light flexible cystoscopy (WLFC) alone in the follow-up strategy for patients after successful initial transurethral resection of bladder cancer to detect recurrence in Sweden. The authors concluded that HAL BLFC allowed more outpatient treatment, improved recurrence detection, reduced transurethral resection of bladder tumors and reduced cystectomies, bed days and operating room time with minimal cost impact across all risk groups. A Markov model was built to investigate the cost of three different follow-up strategies for prostate cancer patients treated with curative intent in the Irish setting [49]. Pearce et al. [49] conducted a cost minimization analysis, and the results were supported by a one-way sensitivity analysis and PSA. They found that the current Irish practice was the least cost-efficient option for prostate cancer follow-up care, while the implementation of alternative models of care such as the NICE guidelines would lead to significant cost savings in the Irish healthcare system. An economic model from Australia compared the implementation of a dietary modification counselling service and individually tailored community-based physical activity programs to a scenario where no lifestyle program is implemented for the survivors of hematological malignancy treated with hemopoietic stem cell transplantation [50]. The authors concluded that the intervention is more likely to be cost-effective for people who were overweight/obese at the baseline. In the USA, a microsimulation model was built to estimate the long-term health and economic outcomes associated with recommended routine cardiography screening for survivors of childhood cancer treated with anthracycline chemotherapy or chest-directed radiotherapy [51]. Childhood cancer survivors who are treated with anthracycline chemotherapy or radiotherapy are at increased risk of developing cardiomyopathy [52]. Ehrhardt et al. [51] found that given the USD 100,000 per QALY gained threshold for cost-effectiveness, screening at 2-, 5- and 10-year intervals appears to be cost-effective for high-risk survivors, and every 5 and 10 years for moderate-risk survivors. Screening every 10 years for low-risk survivors does not appear to be cost-effective. 

### 3.3. Quality Assessment

The methodology for assessing the quality of reporting, presented in Appendix D, describes the quality assessment procedure and the compliance with the CHEERS checklist for each study. As previously mentioned, 14 out of the 34 included studies were published after 2013 when the CHEERS checklist became available [16]. Compliance with the CHEERS checklist ranged from 45 to 98%. One out of the seven studies that achieved more than 90% compliance was published before 2013 [28]. None of the included studies addressed every item listed in the checklist. All studies adequately reported elements relating to background, target population, setting, estimating resources/costs and currency, price date and conversion. For the 16 non-model-based studies, items relating to discount rate, model choice, measurement and valuation of preference-based outcomes, assumptions and description of analytic methods were not applicable. The most poorly reported items related to characterizing uncertainty and heterogeneity. 

Time horizon refers to the period over which costs and outcomes are being evaluated. We included studies that evaluated costs and outcomes for a period of 5 years or more. Less than half (14/34) of the included studies stated why their choice of time horizon was appropriate for the study.

Non-model-based economic evaluations did not apply discounting to costs and health consequences and did not thoroughly describe the underlying assumptions or analytical methods used in the evaluation. Five out of the 18 model-based studies did not apply a discounting rate or did not report the use of a discount rate, with only one study explaining why this was appropriate [32]. Six out of the 13 model-based studies where discount rates were applied did not justify the chosen discount rate. 

For an economic evaluation, effectiveness refers to the ability of an intervention to provide the desired clinical outcome, which is assessed in item 11 of the checklist. Eight studies, including one non-model-based study, met this criterion. The non-model-based study performed a literature search and described the methods used for the identification of the included studies and the synthesis of clinical effectiveness data [20].

### 3.4. Usefulness of Economic Evaluation Studies to Decision-Makers

In assessing the usefulness of the reports, we found that having a high compliance score to the CHEERS reporting checklist does not necessarily guarantee that the study is of great use in decision-making. The summary data extracted in relation to the usefulness of each study are shown in Table 2. Six studies used Markov (state-based) models, one used a decision tree, one study used both a Markov model and a decision tree, while one used discrete event simulation as the model structure. A further six studies used other types of models—namely an empirical model, a validated simulation model, a microsimulation model, a pre-trial economic model and a 5-year projection model. Studies were categorized as having either a “strong”, “moderate” or “limited” level of usefulness for decision-makers. In judging the reporting, we were looking for a clear direction or suggestion about how the results of the analysis could be used to improve the efficiency of healthcare resource use. Seven studies made a clear statement about changing or keeping the allocation of resources or explained how the study’s outcomes are relevant to policies. Ultimately, only one study was rated as “strongly” useful for decision-makers and five studies were rated to be of “moderate” usefulness, while the remaining studies were rated as having “limited” usefulness for decision-makers. The study rated as “strongly” useful was a CUA which utilized a microsimulation model [51], while the studies rated as having “moderate” usefulness were CEAs and CUAs that used a Markov model structure (*n* = 3) [35,40,50], discrete event simulation (*n* = 1) [14] or a 5-year trial model (*n* = 1) [24]. 

## 4. Discussion

The review systematically collated the published economic evaluation studies on long-term cancer survivorship care in OECD countries and identified 34 studies published between January 2000 and November 2020. More than one-third of the included studies evaluated survivorship care for colorectal and breast cancer (13/34). Half of the included studies were modeling studies (16/34). Assessing economic evaluations regarding long-term survivorship care for patients who have had cancer is not an easy task for multiple reasons that limit comparison. Firstly, the technology used for detecting and monitoring recurrence varies widely according to the cancer type that is being recovered from. Secondly, the different follow-up regimens that are possible are also very different according to the type of cancer that has been survived. Thirdly, not all papers report outcomes in the same way, which is an understandable and reasonable difference that exists in this field of research.

Numerous different approaches to providing long-term follow-up were found in our search. Hospital vs. community service utilization, follow-up frequency and adherence to guideline-based follow-up vs. bespoke options were the most commonly identified follow-up regimens that were compared, across all cancer groups. Numerous studies found that reducing the number of follow-up visits did not worsen health outcomes and contributed to a reduction in long-term survivorship care costs. These findings were consistent across various cancer types, including lung [43], cervical [33], skin [30] and breast cancer [27], and make intuitive sense given that increased health service utilization is associated with increased costs, regardless of whether the costing perspective is from the individual, the health system or a third-party payer. A small number of studies suggest that specialist attention is not a cost-effective approach to providing long-term follow-up care in comparison to providing services in primary care by non-specialist medical staff or with less reliance on the heavy use of technological support. Baena-Cañada et al. [34] found that primary care-based services were cost-effective for following up patients with cervical cancer, and Imran et al. [47] found that non-specialist follow-up care was feasible and beneficial from an economic perspective for low-risk thyroid cancer patients, while Shah et al. [37] and Guadagnolo et al. [40] suggest that reducing follow-up intensity by lowering the number of follow-up assessments with PET-CT reduces costs and does not have a detrimental impact on clinical outcomes for patients with head and neck cancers and Hodgkin’s disease, respectively. These findings could be used to support a “less is more” approach to designing follow-up regimens and support the hypothesis that there is possibly an over-servicing associated with long-term follow-up for some cancer survivors.

We found comparison of study results difficult due to the wide variety of methods used and due to differences in how outcomes were reported. A surprising number of studies (*n* = 10) presented results from a costing-only perspective, without the measurement of health effects or outcomes and being identified as only costing studies. A further two studies did not include reference to a clear comparator, meaning that comparative analysis of costs and health effects associated with different approaches to follow-up care was not possible [31,50]. The most commonly reported results were presented as cost per health-related measure (QALY/LYG/HALY) (*n* = 11), followed by cost per follow-up (*n* = 10) and cost per detected recurrence (*n* = 9). Some papers reported more than one outcome of interest. 

Based on the results, it is recommended to conduct model-based economic evaluation studies to support policymakers. As decision-making in healthcare is increasingly including evidence from economic evaluations, we think that the best approach to assess long-term survivorship care for cancer patients is to review the quality of the evidence that is available and assess the quality in the context of what is required for decision-makers to make good decisions. From our review, it is unclear what information is most valued by decision-makers that are tasked with the difficult job of allocating scarce healthcare resources. What is clear is that the majority of studies (26/34) did not provide a clear policy statement regarding resource allocation, leaving decision-makers to interpret the findings in an uncertain manner. We propose that all economic evaluations should include clear and direct statements about how the results should be interpreted and used by decision-makers so that there is no ambiguity regarding the steps that follow. If there are multiple decision-makers—for example, stakeholders who have differing information requirements—we suggest that analysts should provide clear statements regarding the results that fit those information requirements. Put simply, analysts who conduct health economic evaluations must be cognizant of their audience and provide a clear and practical interpretation of their results to support good decision-making in the healthcare setting. 

## 5. Conclusions

Our review shows that there is no shortage of economic evidence relating to long-term cancer survivorship care. All types of economic evaluations, other than cost–benefit analysis, were represented. However, we found that there is a shortage of clear author recommendations that help healthcare decision-makers make decisions about the allocation of scarce resources. Most papers included in the review lacked a clear and practical policy statement, which is a key step for having evidence inform new policies or influence funding allocation. We believe that this issue can be easily corrected if authors more closely adhere to the following steps in future economic evaluations on this topic. First, we recommend that authors follow the CHEERS checklist to ensure that their methods, assumptions and approach are laid bare. This encourages clear and easily digested reporting of the context and methods. Second, we recommend that authors explicitly include a policy statement as part of their presentation of results. Such statements must be clear and direct, acting as recommendations for those charged with putting evidence into practice. Having undertaken this review, we recommend that decision-makers only ever consider economic evaluations that include quality of life data and/or some other relevant patient outcome of interest, so that changes to costs and health outcomes can be assessed. We also recommend that those conducting economic evaluations clearly recommend one of three options for adoption: (1) adopt without delay, (2) do not adopt or (3) design and complete an evaluation plan that allows a clear decision to be made. Finally, based on our review, it is also recommended that policymakers use the findings from decision analytic model-based economic evaluations that are rated as providing “moderately” or “strongly” useful evidence for decision-makers, using criteria that are similar to ours. Following these recommendations will make it easier for decision-makers to use the results for decision-making purposes and bring research findings closer to the decision-making table.

## Figures and Tables

**Table 1:** Descriptive information of included studies.

Descriptive Variable	Number of Studies
Study design	
Retrospective data audit	13
Markov model	9
Other models	6
Randomized control trial	1
Decision tree model	1
Discrete event simulation model	1
Mixed: decision tree + Markov model	1
Quasi-experimental pre/post-study	2
Study Location	
UK	6
USA	5
Germany	4
Australia	4
Canada	4
Italy	3 *
Netherlands	3
Sweden	1
France	1
Finland	1
Ireland	1
Spain	1
Switzerland	1 *
Type of cancer	
Colorectal	7
Breast	6
Cutaneous melanoma	3
Cervical	3
Head and neck	2
Hodgkin’s disease	2
Testicular cancer	2
Prostate	1
Hematological malignancy	1
Bladder	1
Lung	1
Ovarian	1
Renal	1
Thyroid	1
Uterine	1
Not mentioned (childhood cancer)	1
Publication year (CHEERS checklist became available after 2013)	
Before 2013	20
After 2013	14

* One study collected data from participants from both countries, total number exceeds 34; CHEERS—Consolidated Health Economic Evaluation Reporting Standards.

**Table 2:** Usefulness of reviewed studies to decision-making *.

Study	Reasons							Level of Usefulness (Strong/Moderate/Limited)
	Model-Based Design	Applied Model Calibration	Direct and Indirect Costs Included	Quality of Life Measure	Outcome Presented as ICER	Full Sensitivity Analysis (More than Two Combination of OW, MW, PSA, TA and SA)	Policy Suggestion/Direction	
**Colorectal cancer**								
Staib et al. [20]	No	NA	No (direct only)	No	No	NA	No	Limited
Bleeker et al. [18]	No	NA	No (direct only)	No	No	NA	No	Limited
Borie et al. [21]	Yes	No	No (direct only)	Yes	Yes	No (only OW)	Yes	Limited
Renehan et al. [24]	Yes	No	Yes	No	Yes	Yes (OW + SA)	Yes	Moderate
Macafee et al. [22]	Yes	No	No (direct only)	No	No	No (only SA)	No	Limited
Di Cristofaro et al. [19]	No	NA	No (direct only)	No	No	NA	No	Limited
Mant et al. [23]	Yes	No	No (direct only)	Yes	Yes	Yes (SA + TA)	No	Limited
**Breast cancer**								
Grogan et al. [26]	Yes	No	No (direct only)	No	No	Not conducted	No	Limited
Kokko et al. [27]	No	Na	No (direct only)	No	No	NA	No	Limited
Robertson et al. [28]	Yes	No	No (direct only)	Yes	Yes	Yes (OW + MW + TA)	No	Limited
Lu et al. [29]	Yes	No	No (direct only)	No	No	Not conducted	No	Limited
Bessen et al. [14]	Yes	Yes	No (direct only)	Yes	Yes	Yes (SA + PSA)	No	Moderate
Draeger et al. [53]	No	NA	No (direct only)	No	No	NA	No	Limited
**Skin cancer**								
Hengge et al. [30]	Yes	No	No (direct only)	Yes	No	Not conducted	Yes	Limited
Leiter et al. [31]	No	NA	No (direct only)	No	No	NA	No	Limited
Podlipnik et al. [32]	Yes	No	No (direct only)	No	No	Yes (OW + SA)	No	Limited
**Cervical cancer**								
Forni et al. [33]	No	NA	No (direct only)	No	No	NA	No	Limited
Baena-Cañada et al. [34]	No	NA	No (direct only)	Yes	No	NA	No	Limited
Auguste et al. [35]	Yes	No	No (direct only)	Yes	Yes	Yes (OW + SA + PSA)	Yes	Moderate
**Head and neck cancer**								
Shah et al. [37]	No	NA	No (direct only)	No	No	NA	No	Limited
Meregaglia et al. [38]	Yes	No	No (direct only)	Yes	Yes	Yes (OW + TW + PSA)	No	Limited
**Hodgkin’s disease**								
Dryver et al. [39]	No	NA	No (direct only)	No	No	NA	Yes	Limited
Guadagnolo et al. [40]	Yes	No	No (direct only)	Yes	Yes	Yes (OW + SA)	Yes	Moderate
**Testicular cancer**								
Clasen et al. [41]	No	NA	No (direct only)	No	No	NA	No	Limited
Charytonowicz et al. [42]	Yes	No	No (direct only)	No	No	No (only OW)	Yes	Limited
Others								
Gilbert et al. [43]	No	NA	No (direct only)	No	No	NA	No	Limited
Dion et al. [44]	No	NA	No (direct only)	No	No	NA	No	Limited
Rettenmaier et al. [45]	No	NA	No (direct only)	No	No	NA	No	Limited
Rettenmaier et al. [46]	No	NA	No (direct only)	No	No	NA	No	Limited
Imran et al. [47]	No	NA	No (direct only)	No	No	NA	Yes	Limited
Dansk et al. [48]	Yes	No	No (direct only)	No	No	No (only SA)	Yes	Limited
Pearce et al. [49]	Yes	No	No (direct only)	No	No	Yes (OW + PSA)	Yes	Limited
Gao et al. [50]	Yes	No	Yes	Yes	Yes	Yes (OW + PSA)	No	Moderate
Ehrhardt et al. [51]	Yes	Yes	Yes	Yes	Yes	Yes (OW + TW)	Yes	Strong

NOTE. NA: Due to its nature, an item was not relevant to this study. Strong: “Yes” to all items; Moderate: “No” or “NA” or “Not conducted” to one or two items; Limited: “No” or “NA” or “Not conducted” to more than two items. * Adapted from Cheng et al. [8] and McCreanor et al. [9] with copyright permission for use obtained from the corresponding authors. ICER—Incremental Cost-Effectiveness Ratio; OW—One-Way; MW—Multi-Way; PSA—Probabilistic Sensitivity Analysis; TA—Threshold Analysis; SA—Scenario Analysis.

## Appendix A

**Table A1:** PRISMA item checklist.

Section and Topic	Item #	Checklist Item	Reported on Page #
TITLE			
Title	1	Identify the report as a systematic review.	1
ABSTRACT			
Abstract	2	See the PRISMA 2020 for Abstract checklist (Table A2).	1
INTRODUCTION			
Rationale	3	Describe the rationale for the review in the context of existing knowledge.	1
Objectives	4	Provide an explicit statement of the objective(s) or question(s) the review addresses.	1
METHODS			
Eligibility criteria	5	Specify the inclusion and exclusion criteria for the review and how studies were grouped for the syntheses.	2
Information sources	6	Specify all databases, registers, websites, organizations, reference lists and other sources searched or consulted to identify studies. Specify the date when each source was last searched or consulted.	2
Search strategy	7	Present the full search strategies for all databases, registers and websites, including any filters and limits used.	2, Table A3, Appendix B
Selection process	8	Specify the methods used to decide whether a study met the inclusion criteria of the review, including how many reviewers screened each record and each report retrieved, whether they worked independently and, if applicable, details of automation tools used in the process.	2, Appendix B
Data collection process	9	Specify the methods used to collect data from reports, including how many reviewers collected data from each report, whether they worked independently, any processes for obtaining or confirming data from study investigators and, if applicable, details of automation tools used in the process.	2, Appendix B
Data items	10a	List and define all outcomes for which data were sought. Specify whether all results that were compatible with each outcome domain in each study were sought (e.g., for all measures, time points, analyses), and if not, the methods used to decide which results to collect.	2, Appendix B and Appendix C
	10b	List and define all other variables for which data were sought (e.g., participant and intervention characteristics, funding sources). Describe any assumptions made about any missing or unclear information.	2, Appendix B and Appendix C
Study risk of bias assessment	11	Specify the methods used to assess risk of bias in the included studies, including details of the tool(s) used, how many reviewers assessed each study and whether they worked independently, and if applicable, details of automation tools used in the process.	3, Table A6 and Table A7, Appendix D
Effect measures	12	Specify for each outcome the effect measure(s) (e.g., risk ratio, mean difference) used in the synthesis or presentation of results.	NA
Synthesis methods	13a	Describe the processes used to decide which studies were eligible for each synthesis.	2–3
	13b	Describe any methods required to prepare the data for presentation or synthesis, such as handling of missing summary statistics, or data conversions.	2–3, Table A5, Appendix C
	13c	Describe any methods used to tabulate or visually display results of individual studies and syntheses.	2–3, Table A5, Appendix C
	13d	Describe any methods used to synthesize results and provide a rationale for the choice(s). If meta-analysis was performed, describe the model(s), method(s) to identify the presence and extent of statistical heterogeneity and software package(s) used.	2–3
	13e	Describe any methods used to explore possible causes of heterogeneity among study results.	NA
	13f	Describe any sensitivity analyses conducted to assess robustness of the synthesized results.	NA
Reporting bias assessment	14	Describe any methods used to assess risk of bias due to missing results in a synthesis (arising from reporting biases).	3, Table A6 and Table A7, Appendix D
Certainty assessment	15	Describe any methods used to assess certainty (or confidence) in the body of evidence for an outcome.	NA
RESULTS			
Study selection	16a	Describe the results of the search and selection process, from the number of records identified in the search to the number of studies included in the review, ideally using a flow diagram (see Figure 1).	3–11, Table A5, Appendix C and Table A7, Appendix D
	16b	Cite studies that met many but not all inclusion criteria (‘near-misses’) and explain why they were excluded.	Table A4, Appendix B
Study characteristics	17	Cite each included study and present its characteristics.	3–11, Appendix C and Appendix D
Risk of bias in studies	18	Present assessments of risk of bias for each included study.	Appendix D
Results of individual studies	19	For all outcomes, present, for each study: (a) summary statistics for each group (where appropriate) and (b) an effect estimate and its precision (e.g., confidence/credible interval), ideally using structured tables or plots.	3–11, Table A5, Appendix C
Results of syntheses	20a	For each synthesis, briefly summarize the characteristics and risk of bias among contributing studies.	8–11
	20b	Present results of all statistical syntheses conducted. If meta-analysis was done, present for each the summary estimate and its precision (e.g., confidence/credible interval) and measures of statistical heterogeneity. If comparing groups, describe the direction of the effect.	8–11
	20c	Present results of all investigations of possible causes of heterogeneity among study results.	Appendix C and Appendix D
	20d	Present results of all sensitivity analyses conducted to assess the robustness of the synthesized results.	9–11, Table A5, Appendix C
Reporting biases	21	Present assessments of risk of bias due to missing results (arising from reporting biases) for each synthesis assessed.	Table A7, Appendix D
Certainty of evidence	22	Present assessments of certainty (or confidence) in the body of evidence for each outcome assessed.	Table A7, Appendix D
DISCUSSION			
Discussion	23a	Provide a general interpretation of the results in the context of other evidence.	11–12
	23b	Discuss any limitations of the evidence included in the review.	11–12
	23c	Discuss any limitations of the review processes used.	11–12
	23d	Discuss implications of the results for practice, policy and future research.	11–12
OTHER INFORMATION			
Registration and protocol	24a	Provide registration information for the review, including register name and registration number, or state that the review was not registered.	2
	24b	Indicate where the review protocol can be accessed, or state that a protocol was not prepared.	2
	24c	Describe and explain any amendments to information provided at registration or in the protocol.	2
Support	25	Describe sources of financial or non-financial support for the review, and the role of the funders or sponsors in the review.	NA
Competing interests	26	Declare any competing interests of review authors.	12
Availability of data, code and other materials	27	Report which of the following are publicly available and where they can be found: template data collection forms; data extracted from included studies; data used for all analyses; analytic code; any other materials used in the review.	Appendix A, Appendix B, Appendix C and Appendix D

**Table A2:** PRISMA for Abstracts checklist *.

Section and Topic	Item #	Checklist Item	Reported on Page #
TITLE			
Title	1	Identify the report as a systematic review.	1
BACKGROUND			
Objectives	2	Provide an explicit statement of the main objective(s) or question(s) the review addresses.	1
METHODS			
Eligibility criteria	3	Specify the inclusion and exclusion criteria for the review.	1
Information sources	4	Specify the information sources (e.g., databases, registers) used to identify studies and the date when each was last searched.	1
Risk of bias	5	Specify the methods used to assess risk of bias in the included studies.	1
Synthesis of results	6	Specify the methods used to present and synthesize results.	1
RESULTS			
Included studies	7	Give the total number of included studies and participants and summarize relevant characteristics of studies.	1
Synthesis of results	8	Present results for main outcomes, preferably indicating the number of included studies and participants for each. If meta-analysis was performed, report the summary estimate and confidence/credible interval. If comparing groups, indicate the direction of the effect (i.e., which group is favored).	1
DISCUSSION			
Limitations of evidence	9	Provide a brief summary of the limitations of the evidence included in the review (e.g., study risk of bias, inconsistency and imprecision).	1
Interpretation	10	Provide a general interpretation of the results and important implications.	1
OTHER			
Funding	11	Specify the primary source of funding for the review.	NA
Registration	12	Provide the register name and registration number.	1

* This abstract checklist retains the same items as those included in the PRISMA for Abstracts statement published in 2013 but has been revised to make the wording consistent with the PRISMA 2020 statement and includes a new item recommending that authors specify the methods used to present and synthesize results (item #6).

## Appendix B

Review protocol: Economic evaluation of long-term survivorship care for cancer patients in OECD countries: A systematic review

The review was conducted according to the PRISMA 2020 guidelines [11] and included searches of electronic databases and searching of reference lists. It includes original research focusing on economic evaluations for follow-up strategies of patients previously treated for cancer, including screening for certain issues or the cost-effectiveness of long-term follow-up care/survivorship care.

Data sources: Three electronic databases, namely Medline, PubMed and PsycINFO, and the National Health Service Economic Evaluation Databases as well as the Health Technology Assessment Databases were searched with the assistance of librarians.

**Table A3:** Search strategy.

	Search Terms	Numbers
PUBMED		
1.	“economic evaluation*” or “economic analys*” or “cost*” or expenditure or “out of pocket” or “cost of illness*” or “health care cost*” or “direct service cost*” or “drug cost*” or “hospital cost*”	883,278
2	“Costs and Cost Analysis” [MeSH Terms] OR “Economics” [MeSH Terms] OR “Economics” [MeSH Subheading] OR “Cost of Illness” [MeSH Terms] OR “Cost Sharing” [MeSH Terms] OR “Cost Savings” [MeSH Terms] OR “technology, high cost” [MeSH Terms] OR “Cost Control” [MeSH Terms] OR “Cost-Benefit Analysis” [MeSH Terms] OR “Cost Allocation” [MeSH Terms] OR “Health Care Costs” [MeSH Terms] OR “Direct Service Costs” [MeSH Terms] OR “Hospital Costs” [MeSH Terms] OR “Employer Health Costs” [MeSH Terms] OR “Drug Costs” [MeSH Terms] OR “Health Expenditures” [MeSH Terms]	742,199
3	1 OR 2	1,328,609
4	“follow-up” or “secondary prevent*” or “after treatment” or “after chemo*” or “after radiation” or “after care” or “after cur*” or “post treatment” or “post chemo*” or “post radiotherapy” or “survivorship care” or “long-term strateg*”	1,568,415
5	“Neoplasms” [MeSH Terms]	3,378,598
6	(cancer* or carcinoma* or histiocytosis or leukemia or lymphoma* or medulloblastoma* or neoplasm* or nephroblastoma* or neuroblastoma* or oncolog* or osteosarcoma* or retinoblastoma* or sarcoma* or tumor* or neoplasm*).ti,ab.	3,448,803
7	5 OR 6	4,370,095
8	(Australia or Austria or Belgium or Canada or Chile or Colombia or Czech Republic or Denmark or Estonia or Finland or France or Germany or Greece or Hungary or Iceland or Ireland or Israel or Italy or Japan or Korea or Latvia or Lithuania or Luxembourg or Mexico or Netherlands or “New Zealand” or Norway or Poland or Portugal or Slovak Republic or Slovenia or Spain or Sweden or Turkey or “United Kingdom” or England or United States).ti,ab.	1,153,236
9	3 and 4 and 7 and 8	1499
10	Filters: 2000–2020 and English	1277
2. MEDLINE		
	(“economic evaluation*” or “economic analys*” or “cost* utility” or “cost analysis” or “cost effective*” or “cost benefit” or “cost minimization” or “cost minimization” or expenditure or “out of pocket” or “cost of illness*” or “health care cost*” or “direct service cost*” or “drug cost*” or “hospital cost*”) AND (“Follow-up” or “secondary prevent*” or “after treatment” or “after chemo*” or “after radiation” or “after care” or “after cur*” or “post treatment” or “post chemo*” or “post radiotherapy” or “survivorship care” or “long-term strateg*” or “short-term strateg*”) AND (Cancer* or carcinoma* or histiocytosis or leukemia or lymphoma* or medulloblastoma* or neoplasm* or nephroblastoma* or neuroblastoma* or oncolog* or osteosarcoma* or retinoblastoma* or sarcoma* or tumor* or neoplasm*) AND (Australia or Austria or Belgium or Canada or Chile or Colombia or Czech Republic or Denmark or Estonia or Finland or France or Germany or Greece or Hungary or Iceland or Ireland or Israel or Italy or Japan or Korea or Latvia or Lithuania or Luxembourg or Mexico or Netherlands or “New Zealand” or Norway or Poland or Portugal or Slovak Republic or Slovenia or Spain or Sweden or Turkey or “United Kingdom” or England or United States)Filters: 2000–2020 and English	2107
3. PsychINFO		
	(“economic evaluation*” or “economic analys*” or “cost* utility” or “cost analysis” or “cost effective*” or “cost benefit” or “cost minimization” or “cost minimization” or expenditure or “out of pocket” or “cost of illness*” or “health care cost*” or “direct service cost*” or “drug cost*” or “hospital cost*”) AND (“follow-up” or “secondary prevent*” or “after treatment” or “after chemo*” or “after radiation” or “after care” or “after cur*” or “post treatment” or “post chemo*” or “post radiotherapy” or “survivorship care” or “long-term strateg*” or “short-term strateg*”) AND (cancer* or carcinoma* or histiocytosis or leukemia or lymphoma* or medulloblastoma* or neoplasm* or nephroblastoma* or neuroblastoma* or oncolog* or osteosarcoma* or retinoblastoma* or sarcoma* or tumor* or neoplasm*) AND (Australia or Austria or Belgium or Canada or Chile or Colombia or Czech Republic or Denmark or Estonia or Finland or France or Germany or Greece or Hungary or Iceland or Ireland or Israel or Italy or Japan or Korea or Latvia or Lithuania or Luxembourg or Mexico or Netherlands or “New Zealand” or Norway or Poland or Portugal or Slovak Republic or Slovenia or Spain or Sweden or Turkey or “United Kingdom” or England or United States)Filters: 2000–2020 and English	204
4. National Health Service Economic Evaluation Databases up to 2015		
	(“Follow-up” or “secondary prevent*” or “after treatment” or “after chemo*” or “after radiation” or “after care” or “after cur*” or “post treatment” or “post chemo*” or “post radiotherapy” or “survivorship care” or “long-term strateg*” or “short-term strateg*”) AND (Cancer* or carcinoma* or histiocytosis or leukemia or lymphoma* or medulloblastoma* or neoplasm* or nephroblastoma* or neuroblastoma* or oncolog* or osteosarcoma* or retinoblastoma* or sarcoma* or tumor* or neoplasm*) AND (Australia or Austria or Belgium or Canada or Chile or Colombia or Czech Republic or Denmark or Estonia or Finland or France or Germany or Greece or Hungary or Iceland or Ireland or Israel or Italy or Japan or Korea or Latvia or Lithuania or Luxembourg or Mexico or Netherlands or “New Zealand” or Norway or Poland or Portugal or Slovak Republic or Slovenia or Spain or Sweden or Turkey or “United Kingdom” or England or United States)Filters: English	750
5. National Health Service Economic Evaluation Databases		
	(“follow-up” or “secondary prevent*” or “after treatment” or “after chemo*” or “after radiation” or “after care” or “after cur*” or “post treatment” or “post chemo*” or “post radiotherapy” or “survivorship care” or “long-term strateg*” or “short-term strateg*”) AND (cancer* or carcinoma* or histiocytosis or leukemia or lymphoma* or medulloblastoma* or neoplasm* or nephroblastoma* or neuroblastoma* or oncolog* or osteosarcoma* or retinoblastoma* or sarcoma* or tumor* or neoplasm*) AND (Australia or Austria or Belgium or Canada or Chile or Colombia or Czech Republic or Denmark or Estonia or Finland or France or Germany or Greece or Hungary or Iceland or Ireland or Israel or Italy or Japan or Korea or Latvia or Lithuania or Luxembourg or Mexico or Netherlands or “New Zealand” or Norway or Poland or Portugal or Slovak Republic or Slovenia or Spain or Sweden or Turkey or “United Kingdom” or England or United States)Filters: English	67
6. From other source: reference list review of any article chosen for possible inclusion		17

**Question of interest:** What is the current cost-effectiveness evidence for long-term survivorship care compared to usual care for cancer patients?

**Cancer survivorship:** The most widely used definition of cancer survivorship is from the National Coalition for Cancer Survivorship in the USA and includes for each person the period “from the time of diagnosis, through the balance of his or her life, regardless of the ultimate cause of death” [2].

**Stages of survivorship:** Different stages of survivorship comprise acute (diagnosis to treatment), chronic (ongoing) and long-term/late survivorship (≥5 years post-diagnosis) [3].

**Population:** Cancer patients of any age and sex who have received survivorship care after initial cancer treatment.

**Intervention(s), exposure(s):**

− Any long-term strategy or any long-term follow-up care or survivorship care (≥5 years after initial treatment) as care used in a supportive role to improve quality of life as well as diagnostic strategy to detect recurrence. Types of care that were included in the review were:
  ○ Management and/or screening for certain issues such as curable recurrence
  ○ Diets, including the use of dietary supplements
  ○ Exercise
  ○ Counseling
  ○ E-health technologies: web-based or app-based e-health interventions
− Fertility treatments
− Any study setting (e.g., hospital- or community-based)
− Any form of recruitment

**Comparator(s)/control:** Any control group or comparators assigned when comparing an intervention or strategy related to the strategy for cancer survivors as well as no comparator if it is a costing-only study.

**Study designs of interest:** Prospective and retrospective cohorts, randomized control trials and economic modeling studies.

**Inclusion criteria:**

1. Studies that either estimated the impact of survivorship care, comparing it to no survivorship care, or compared different long-term care strategies for cancer survivors in OECD countries
2. Economic evaluations: costing studies, cost-effectiveness analyses, cost–utility analyses, cost–consequences analyses and cost minimization analyses were included if they reported both the costs and benefits expected for both usual care and the comparator(s)
3. Research articles published in English language
4. Research limited to human studies
5. Full publication or manuscript available for review

**Exclusion criteria:**

Articles were initially excluded if they were duplicates or if the title clearly demonstrated that the intervention and outcome of interest were not the focus of the review. Articles were then excluded based on the following:

1. Economic evaluation of active surveillance vs. initial treatment (5)
2. Economic evaluation of curative interventions (7)
3. Economic evaluation of follow-up care for hyperplasia/dysplasia (12)
4. Economic evaluation of follow-up care for mixed patients (1)
5. Economic evaluation of management of chemo/radiotherapy-induced symptoms (22)
6. Economic evaluation of palliative care (2)
7. Economic evaluation of supporting management during initial treatment (31)
8. Not follow-up/survivorship care (2)
9. Not economic study (4)
10. Only focused on costs and did not report health benefits (34)
11. Protocols/conference abstract/review papers (38)
12. Used same data/reported same study (4)
13. Economic evaluation of breast implant (4)
14. Economic issue of survivors (8)
15. Followed up for less than 5 years (27)

**Table A4:** A list of studies that met many inclusion criteria (“near-misses”) but were excluded due to a short follow-up period (less than 5 years).

#	Study Author and Publication Year	Follow-Up Period
Colorectal cancer		
1	Augestad et al. [54]	24 months
2	Verberne et al. [55]	3 years
Breast cancer		
3	Beaver et al. [56]	24 months
4	Benning et al. [57]	12 months
5	Burm et al. [58]	9–15 months
6	Coyle et al. [59]	24 months
7	Kimman et al. [60]	1 year
8	Oltra et al. [61]	3 years
9	Wojcinski et al. [62]	12 months
Ovarian, uterine cancer		
10	Armstrong et al. [63]	2 years
11	Dixon et al. [64]	12 months
Head and neck cancer		
12	Ham et al. [65]	3.5 months
Others		
13	Bongers et al. [66]	Mean (SD) = 31.6 (9.8) months
14	Greuter et al. [67]	3 and 6 months
15	Heinzel et al. [68]	Unclear
16	Jeyarajah et al. [69]	Mixed 3 and/or 5 years
17	Kampshoff et al. [70]	12 weeks
18	Kent et al. [71]	Up to 5 years
19	Lizée et al. [72]	2 years
20	Moore et al. [73]	12 months
21	Nam et al. [74]	Unclear
22	Polinder et al. [75]	12 months
23	Pollack et al. [76]	366–1095 days
24	Shih et al. [77]	6 months
25	van der Spek et al. [78]	6 months
26	van Dongen et al. [79]	12 months
27	van Loon et al. [80]	Up to 5 years

**Main outcome(s):** Assess the cost-effectiveness of long-term survivorship care for cancer survivors.

**Measures of effect:** Incremental effectiveness, incremental costs and ICER (incremental cost-effectiveness ratio) values.

**Data extraction:** The initial search was performed by two reviewers using pre-determined search terms and strategies from chosen databases. All identified papers were imported into EndNote and screened in accordance with PRISMA guidelines.

After removal of duplicates, the titles and abstracts were screened for relevance and eligibility criteria. The two reviewers then extracted the data from the studies selected for inclusion using a pre-designed extraction form. The data extraction sheet was first pilot tested on 10 studies and then revised accordingly to include the following.

**Identification of study:**

16. Record the first author’s last name and initials
17. Record the journal name
18. Record the year of publication
19. Record the volume and page numbers

**Characteristics of study:**

20. Setting
21. Type of cancer
22. Patient population (age if available)
23. Intervention
24. Comparator
25. Type of economic evaluation
26. Study design
27. Discount rate
28. Perspective
29. Costs included
30. Time horizon/study period
31. Outcome measures
32. Baseline analysis
33. Sensitivity analysis
34. Main results
35. Additional comments (a threshold value, etc.)

**Quality of reporting assessment:** The full texts of all included articles were assessed for reporting quality by two independent reviewers using the Consolidated Health Economic Evaluation Reporting Standards (CHEERS) checklist. Any discrepancies over reporting quality assessment between the two reviewers were resolved by discussion with a third reviewer. The standards of the input data for health economic analysis in the decision model were ranked based on the hierarchy adapted by Cooper and colleagues.

**Strategy for data synthesis:** After screening the title, abstract and full text, data were extracted from relevant articles and were summarized in tables. The data fields were as follows: research question, setting and location, perspective, time horizon, discount rate, structure of the economic model if applied, study population, intervention and comparator, outcome measures, incremental cost-effectiveness ratio (ICER) and sensitivity analysis method.

The quality of the selected economic evaluations was assessed using the Consolidated Health Economic Evaluation Reporting Standards (CHEERS) checklist. This checklist provides 24 items, with accompanying recommendations and examples to ensure more consistency and transparent reporting of economic evaluations. Each item in the CHEERS checklist was scored as having met the criteria in full (1), partially (0.5) or not at all (0) or as not applicable (NA).

## Appendix C

**Table A5:** Characteristics of included studies.

	Author (Publication Year)	Setting	Type of Cancer	Intervention	Comparator	EE Type	Study Design	Discount Rate	Perspective	Costs Included	Time Horizon Study Period	Outcome Measures	Sensitivity Analysis
1	Staib et al., (2000)	Germany	Colorectal	Intensive follow-up	None	Costing study	Retrospective data audit	NA	Not reported (health system)	Personnel, infrastructure and test costs	10 years	Cost per followed cancer patient	Not conducted
2	Bleeker et al., (2001)	The Netherlands	Colonic	Mixed follow-up	None	Costing study	Retrospective data audit	NA	Not reported (health system)	Tests and examination costs	43 months	Cost of follow-up diagnostic event per curative resected recurrence	Not conducted
3	Borie et al., (2004)	France	Colorectal	Standard follow-up	Simplified follow-up	CUA	Markov Model	NA	Not reported (health system)	The costs of the examination carried out	5-7 years	Δcost/ΔQALY	OW
4	Renehan et al., (2004)	UK	Colorectal	Intensive follow-up	Usual follow-up	CEA	5-year trail model	Benefits 1.5% and costs 6%	Health service perspective	Direct, indirect and overhead costs	5 years	Δcost/ΔLY	OW, SA
5	Macafee et al., (2008)	UK	Colorectal	Intensive follow-up	Usual follow-up	CEA	Retrospective data used for 5-year projection	Costs 3.5%	Hospital perspective	Direct hospital costs	5 years	Cost of follow-up and cost of resectable recurrence	SA
6	Di Cristofaro et al., (2012)	Italy	Colorectal	Multiple surveillance protocols	None	Costing study	Retrospective data audit	NA	Not reported (health system)	Costs for follow-up tests	5 years	The costs and the percentage of recurrence following the various surveillance protocols Recurrence rate	Not conducted
7	Mant et al., (2017)	UK	Colorectal	CT and CEA follow-up	Minimal follow-up	CUA	Randomized controlled trial and a pre-trial economic model	Costs and benefits 3.5%	The perspective of the UK NHS	Costs for visits and tests	8 years	Δcost/ΔQALY	SA, TA
8	Grogan et al., (2002)	Australia	Breast	13 follow-up schedules	Minimal follow-up	Costing study	Retrospective data audit used to establish an empirical model	NA	Not reported (health system)	Costs for visits and tests	70 months	Cost of follow-up per detection of salvageable event per patient	Not conducted
9	Kokko et al., (2005)	Finland	Breast	Routine follow-up	Unclear	Costing study	Randomized controlled trial	No	The hospital perspective	Costs for visits and tests	4 years	Cost of follow-up per patient Cost per detected recurrence	Not conducted
10	Robertson et al., (2011)	UK	Breast	Mammography	No surveillance	CUA	Markov modeling	Costs and benefits 3.5%	The UK NHS	Costs incurred by the NHS	Lifetime	Δcost/ΔQALY	OW, MW, TA
11	Lu et al., (2012)	The Netherlands	Breast	3 alternate strategies	Guideline follow-up	CEA	An extended and validated simulation model	NA	Not reported (health system)	Costs for follow-up tests Percentage of small tumors identified by tests	Lifetime	Detection rate of small tumors (2 cm or smaller) and associated costs for each strategy Additional costs associated with an increase of 1% in the number of early breast cancers detected	Not conducted
12	Bessen et al., (2015)	Australia	Breast	Intensive follow-up	Simplified follow-up	CUA	Retrospective data audit and discrete event simulation model	NA	Not reported (health system)	Costs for tests	5 years	Cost per QALY gained; Δcost/ΔQALY	SA, PSA
13	Draeger et al., (2020)	The Netherlands	Breast	Unclear	Unclear	CEA	Quasi-experimental pre/post study	NA	Not reported (health system)	Costs for diagnostic procedures, clinical follow-up visits	5 years	Potential savings of follow-up	Predefined follow-up sensitivity analysis
14	Hengge et al., (2007)	Germany	Melanoma	Intensive follow-up	Guideline follow-up	CUA	Markov model	NA	Not reported (health system)	Actual costs for materials, scans, human resources and overhead costs	5 years	Costs per detected metastasis and cost per QALY gained	Not conducted
15	Leiter et al., (2009)	Germany	Melanoma	Technical	None	CEA	Retrospective data audit	NA	Not reported (health system)	Cost for each technical follow-up investigation	5 years	Costs for the detection of one recurrence	Not conducted
16	Podlipnik et al., (2019)	Spain	Malignant melanoma	CT	Contrast brain MRI	CEA	Decision tree	Not applied	Healthcare system	Costs for visits and tests	5 years	Cost-effectiveness ratio per patient	OW, SA
17	Forni et al., (2007)	Italy	Cervical	Simplified follow-up	Usual follow-up	CEA	Retrospective data audit	NA	Not reported (health system)	The costs of the examination carried out	5 years	The number of recurrences, cost of per recurrence per patient	Not conducted
18	Baena-Cañada et al., (2013)	UK	Cervical	Primary care follow-up	Specialist-led follow-up	CMA	Retrospective data audit	NA	Not reported (health system)	Costs for visits and complementary tests	5 years	Cost of the follow-up, events, HRQL and satisfaction	Not conducted
19	Auguste et al., (2014)	UK	Cervical	MRI with or without CT	Clinical follow-up	CUA	Markov model	Costs 3.5%	Healthcare system	Costs for tests	5 years	Δcost/ΔQALY	OW, SA, PSA
20	Shah et al., (2015)	Australia	Head, neck + nasopharyngeal	PET-CT scan	No PET-CT scan	CEA	Quasi-experimental pre/post study	Costs 5%	Hospital perspective	Direct costs	5 years	Cost of follow-up strategies The proportion of radically treatable recurrences	Not conducted
21	Meregaglia et al., (2018)	Italy and Switzerland	Head and neck	Intensive follow-up	Symptom-driven surveillance	CUA	Markov model	Costs and benefits 3%	Healthcare system	Costs for hospital admissions, specialist visits, radiological exams, laboratory tests and outpatient treatment	Lifetime	Δcost/ΔQALY Δcost/ΔLYG	OW, TW, PSA
22	Dryver et al., (2003)	Canada	Hodgkin’s disease	CXR, CT, blood count	None	Costing study	Retrospective data audit	NA	Not reported (health system)	Costs for visits and tests	1–120 months	the cost per true relapse	Not conducted
23	Guadagnolo et al., (2006)	USA	Hodgkin’s disease	Routine annual CT	Non-CT modalities	CUA	Markov model	Costs and benefits 3%	Modified societal perspective	Visits and blood tests	Lifetime	Δcost/ΔQALY and Δcost/ΔLY	SA, OW
24	Clasen et al., (2009)	Germany	Seminoma	Technical	None	Costing study	Retrospective data audit	NA	Not reported (health system)	Cost for each technical follow-up investigation	10 years	Cost per relapse detected	Not conducted
25	Charytonowicz et al., (2019)	USA	TGCT	mRNA testing	CT-based follow-up	Costing study	Markov model	Costa and benefits 5%	Healthcare system	Costs for visits and tests	10 years	The sensitivity of each tests The cost of follow-up care	OW
26	Gilbert et al., (2000)	Canada	Lung	Specialist outpatient	Non-specialist follow-up	CEA	Retrospective data audit	NA	Not reported (health system)	Personnel, infrastructure and test costs	1 to 107 months	Cost per recurrence detected by a surgeon or FP and 5 years survival rate	Not conducted
27	Dion et al., (2010)	Canada	Renal	Clinical guidelines	Usual follow-up	CEA	Retrospective data audit	NA	Not reported (health system)	Costs for visits and tests	5 years	The total cost of follow-up per patient and per patient month The cost per recurrence	Not conducted
28	C. R. Rettenmaier et al., (2010)	USA	Uterine	Imaging	None	Costing study	Retrospective data audit	NA	Not reported (health system)	Costs for tests and visits	20 years	The cost per patient recurrence	Not conducted
29	N. Rettenmaier et al., (2010)	USA	Ovarian and primary peritoneal	Imaging	None	Costing study	Retrospective data audit	NA	Not reported (health system)	Costs for tests and visits	16 years	The cost per patient recurrence	Not conducted
30	Imran et al., (2019)	Canada	Thyroid	Primary care follow- up	Tertiary care follow-up	CEA	Retrospective data audit	NA	Not reported	Costs for visits and tests Travel costs	62 months	Cost of follow-up care and travel cost per patients Rates of recurrence and cancer-related mortality	Not conducted
31	Dansk et al., (2016)	Sweden	Bladder	Flexible cystoscopy with WLFC and BLFC	Flexible cystoscopy with WLFC only	CCA	Mixed: decision tree and Markov model	Costs 3%	Hospital and other purchaser perspectives	Costs for visits and tests	5 years	Cost of follow-up strategy Detection rate, hospital bed days and number of procedures	SA
32	Pearce et al., (2016)	Ireland	Prostate	Guideline follow-up	Current guideline	CMA	Markov model	Costs 5%	Healthcare payer	Costs for visits	10 years	The average cost of follow-up per survivor	OW, PSA
33	Gao et al., (2017)	Australia	Hem. malignancy	12-month intervention	None	CEA	Markov model	Costs and benefits 3%	Health sector perspective	Costs for individual fitness activities, dietician time, project manager/physiotherapist time	Lifetime	Δcost/Δhealth- adjusted life years (HALYs) gained	OW, SA
34	Ehrhardt et al., (2020)	USA	Childhood cancer	1-, 2-, 3-, 5- and 10-year interval screening	No screening	CUA	Microsimulation model	Costs and benefits 3%	Not reported (societal perspective)	Medical costs and indirect patient costs, such as lost work time	Lifetime	Δcost/ΔQALY	OW, TW

CUA—cost–utility analysis; CEA—cost-effectiveness analysis; CCA—cost–consequence analysis; CMA—cost minimization analysis; OW—one-way; PSA—probabilistic sensitivity analysis; SA—scenario analysis; TA—threshold analysis; MW—multi-way; QALY—quality-adjusted life year; HALY—health-adjusted life year; LYG—life-year gained.

## Appendix D

**Table A6:** CHEERS checklist—items to include when reporting economic evaluation of health interventions.

Number of ITEM	Description of ITEM	Summary of Criterion
Item 1	Identify the study as an economic evaluation or use more specific terms such as “cost-effectiveness analysis”, and describe the interventions compared.	Title identifies an economic evaluation
Item 2	Provide a structured summary of objectives, perspective, setting, methods (including study design and inputs), results (including base case and uncertainty analyses) and conclusions.	Structured summary was presented in abstract
Item 3	Provide an explicit statement of the broader context for the study. Present the study question and its relevance for health policy or practice decisions.	Background and objectives were provided
Item 4	Describe characteristics of the base case population and subgroups analyzed, including why they were chosen.	Target population and subgroups were reported
Item 5	State relevant aspects of the system(s) in which the decision(s) need(s) to be made.	Setting and location were reported
Item 6	Describe the perspective of the study and relate this to the costs being evaluated.	Study perspective was reported
Item 7	Describe the interventions or strategies being compared and state why they were chosen.	Comparator(s) was/were reported
Item 8	State the time horizon(s) over which costs and consequences are being evaluated and say why appropriate.	Time horizon was reported
Item 9	Report the choice of discount rate(s) used for costs and outcomes and say why appropriate.	Discount rate was used
Item 10	Describe what outcomes were used as the measure(s) of benefit in the evaluation and their relevance for the type of analysis performed.	Choice of health outcomes was reported
Item 11	a. Single study-based estimates: Describe fully the design features of the single effectiveness study and why the single study was a sufficient source of clinical effectiveness data. b. Synthesis-based estimates: Describe fully the methods used for identification of included studies and synthesis of clinical effectiveness data.	a. Measurement of effectiveness (single study-based) b. Measurement of effectiveness (synthesis-based) was reported
Item 12	If applicable, describe the population and methods used to elicit preferences for outcomes.	Measurement and valuation of preference-based outcomes
Item 13	a. Single study-based economic evaluation: Describe approaches used to estimate resource use associated with the alternative interventions. Describe primary or secondary research methods for valuing each resource item in terms of its unit cost. Describe any adjustments made to approximate to opportunity costs. b. Model-based economic evaluation: Describe approaches and data sources used to estimate resource use associated with model health states. Describe primary or secondary research methods for valuing each resource item in terms of its unit cost. Describe any adjustments made to approximate to opportunity costs.	a. Estimating resources and costs—single study-based b. Estimating resources and costs—model-based
Item 14	Report the dates of the estimated resource quantities and unit costs. Describe methods for adjusting estimated unit costs to the year of reported costs if necessary. Describe methods for converting costs into a common currency base and the exchange rate.	Currency, price date and conversion were reported
Item 15	Describe and give reasons for the specific type of decision-analytical model used. Providing a figure to show model structure is strongly recommended.	Choice of model was reported
Item 16	Describe all structural or other assumptions underpinning the decision-analytical model.	Assumptions were described
Item 17	Describe all analytical methods supporting the evaluation. This could include methods for dealing with skewed, missing, or censored data; extrapolation methods; methods for pooling data; approaches to validate or make adjustments (such as half cycle corrections) to a model; and methods for handling population heterogeneity and uncertainty.	Analytic methods were described
Item 18	Report the values, ranges, references and, if used, probability distributions for all parameters. Report reasons or sources for distributions used to represent uncertainty where appropriate. Providing a table to show the input values is strongly recommended.	Study parameters were reported
Item 19	For each intervention, report mean values for the main categories of estimated costs and outcomes of interest, as well as mean differences between the comparator groups. If applicable, report incremental cost-effectiveness ratios.	Incremental cost and outcomes were reported
Item 20	a. Single study-based economic evaluation: Describe the effects of sampling uncertainty for the estimated incremental cost and incremental effectiveness parameters, together with the impact of methodological assumptions (such as discount rate, study perspective). b. Model-based economic evaluation: Describe the effects on the results of uncertainty for all input parameters, and uncertainty related to the structure of the model and assumptions.	a. Characterizing uncertainty (single study-based) b. Characterizing uncertainty (model-based) was reported
Item 21	If applicable, report differences in costs, outcomes, or cost-effectiveness that can be explained by variations between subgroups of patients with different baseline characteristics or other observed variability in effects that are not reducible by more information.	Characterizing heterogeneity was reported
Item 22	Summarize key study findings and describe how they support the conclusions reached. Discuss limitations and the generalizability of the findings and how the findings fit with current knowledge.	Study findings, limitations, generalizability and current knowledge were mentioned
Item 23	Describe how the study was funded and the role of the funder in the identification, design, conduct, and reporting of the analysis. Describe other nonmonetary sources of support.	Source of funding was mentioned
Item 24	Describe any potential for conflict of interest of study contributors in accordance with journal policy. In the absence of a journal policy, we recommend authors comply with International Committee of Medical Journal Editors recommendations.	Conflicts of interest were presented

The reporting quality of the selected papers was assessed using the Consolidated Health Economic Evaluation Reporting Standards (CHEERS), consisting of a 24-item checklist with accompanying recommendations to ensure consistent and transparent reporting in economic evaluations [16].

**Table A7:** Quality of reporting assessment using the CHEERS criteria.

			Item 1	Item 2	Item 3	Item 4	Item 5	Item 6	Item 7	Item 8	Item 9	Item 10	Item 11	Item 12	Item 13	Item 14	Item 15	Item 16	Item 17	Item 18	Item 19	Item 20	Item 21	Item 22	Item 23	Item 24
	Summary of criterion	Compliance with the CHEERS checklist	Title identifies an economic evaluation	Structured summary was presented in abstract	Background and objectives were provided	Target population and subgroups were reported	Setting and location were reported	Study perspective was reported	Comparator(s) was/were reported	Time horizon was reported	Discount rate was used	Choice of health outcomes was reported	a. Measurement of effectiveness (single study-based)b. Measurement of effectiveness (synthesis-based) was reported	Measurement and valuation of preference-based outcomes	a. Estimating resources and costs (single study-based)b. Estimating resources and costs (model-based)	Currency, price date and conversion were reported	Choice of model was reported	Assumptions were described	Analytic methods were described	Study parameters were reported	Incremental cost and outcomes were reported	a. Characterizing uncertainty (single study-based)b. Characterizing uncertainty (model based) was reported	Characterizing heterogeneity was reported	Study findings, limitations, generalizability and current knowledge were mentioned	Source of funding was mentioned	No conflicts of interest were presented
**Colorectal cancer**																										
1	Staib et al., (2000)	50%	1	0.5 ^b^	1	1	1	0	0	1	NA	1	1	NA	1	1	NA	NA	NA	0	0	0	0	0.5 ^i^	0	0
2	Bleeker et al., (2001)	48%	0.5 ^a^	0.5 ^b^	1	1	1	0	0	0.5 ^c^	NA	1	0.5 ^e^	NA	1	1	NA	NA	NA	0	0	0	0	0.5 ^i^	1	0
3	Borie et al., (2004)	65%	1	0.5 ^b^	1	1	1	0	0.5 ^c^	1	0	1	0.5 ^e^	1	1	1	1	0	1	0	1	0	1	1	0	0
4	Renehan et al., (2004)	83%	1	1	1	1	1	1	1	1	0.5 ^c^	1	0.5 ^e^	NA	1	1	1	1	1	0	1	0	0	1	1	1
5	Macafee et al., (2008)	63%	1	0.5 ^b^	1	1	1	1	1	0.5 ^c^	0.5 ^c^	1	0.5 ^e^	NA	1	1	0	1	0	0	0	0	1	1	0	1
6	Di Cristofaro et al., (2012)	60%	1	0.5 ^b^	1	1	1	0	0	0.5 ^c^	NA	1	0.5 ^e^	NA	1	1	NA	NA	NA	1	0	1	1	0.5 ^i^	0	0
7	Mant et al., (2017)	85%	1	0.5 ^b^	1	1	1	1	1	1	0.5 ^c^	1	0.5 ^e^	1	1	1	0.5 ^f^	1	1	1	1	0.5 ^h^	0	1	1	1
**Breast cancer**																										
8	Grogan et al., (2002)	46%	0.5 ^a^	0.5 ^b^	1	1	1	0	1	0.5 ^c^	0	1	0.5 ^e^	NA	1	1	0.5 ^f^	0	0	0	0	0	0	0.5 ^i^	1	0
9	Kokko et al., (2005)	60%	0.5 ^a^	0.5 ^b^	1	1	1	1	1	0.5 ^c^	NA	1	0.5 ^e^	NA	1	1	NA	NA	NA	0	0	0	0	1	1	0
10	Robertson et al., (2011)	92%	1	0.5 ^b^	1	1	1	1	1	0.5 ^c^	1	1	1	1	1	1	1	1	1	1	1	0	1	1	1	1
11	Lu et al., (2012)	65%	1	0.5 ^b^	1	1	1	0	1	1	0	1	0.5 ^e^	NA	1	1	1	0	0.5 ^g^	1	0	1	0	1	0	1
12	Bessen et al., (2015)	71%	1	0.5 ^b^	1	1	1	0	1	0.5 ^c^	0	1	0.5 ^e^	NA	1	1	1	1	1	1	0	1	1	1	1	0
13	Draeger et al., (2020)	68%	0.5 ^a^	0.5 ^b^	1	1	1	0	1	1	NA	1	0.5 ^e^	NA	1	1	NA	NA	NA	0	1	0	0	1	1	1
**Skin cancer**																										
14	Hengge et al., (2007)	52%	1	0.5 ^b^	1	1	1	0	1	0.5 ^c^	0	1	0.5 ^e^	NA	1	1	1	0	0	0	0	0	1	0	0	1
15	Leiter et al., (2009)	58%	0.5 ^a^	0.5 ^b^	1	1	1	0	0	1	NA	1	0.5 ^e^	NA	1	1	NA	NA	NA	0	0	1	1	0	1	0
16	Podlipnik et al., (2019)	71%	1	0.5 ^b^	1	1	1	1	1	0.5 ^c^	0.5 ^d^	1	0.5 ^e^	NA	1	1	1	0	1	0	1	0	0	1	1	1
**Cervical cancer**																										
17	Forni et al., (2007)	53%	1	0.5 ^b^	1	1	1	0	1	0.5 ^c^	NA	1	0.5 ^e^	NA	1	1	NA	NA	NA	0	0	0	0	1	0	0
18	Baena-Cañada et al., (2013)	60%	0	0	1	1	1	0	1	0.5 ^c^	NA	1	0.5 ^e^	1	1	1	NA	NA	NA	0	0	0	0	1	1	1
19	Auguste et al., (2014)	94%	1	0.5 ^b^	1	1	1	1	1	1	1	1	1	1	1	1	1	1	1	1	1	1	0	1	1	1
**Head and neck cancer**																										
20	Shah et al., (2015)	66%	0.5 ^a^	0.5 ^b^	1	1	1	1	1	1	1	1	0.5 ^e^	NA	1	1	NA	1	NA	0	0	0	0	1	0	1
21	Meregaglia et al., (2018)	90%	1	0.5 ^b^	1	1	1	1	1	0.5 ^c^	0.5 ^c^	1	1	1	1	1	1	1	1	1	1	1	0	1	1	1
**Hodgkin’s disease**																										
22	Dryver et al., (2003)	48%	0.5 ^a^	0.5 ^b^	1	1	1	0	0	1	NA	1	0.5 ^e^	NA	1	1	NA	NA	NA	0	0	0	0	1	0	0
23	Guadagnolo et al., (2006)	88%	1	0.5 ^b^	1	1	1	1	1	0.5 ^c^	1	1	1	1	1	1	1	1	1	1	1	0	0	1	1	1
**Testicular cancer**																										
24	Clasen et al., (2009)	48%	0.5 ^a^	0	1	1	1	0	0	0.5 ^c^	NA	1	0.5 ^e^	NA	1	1	NA	NA	NA	0	0	0	1	1	0	0
25	Charytonowicz et al., (2019)	79%	1	0.5 ^b^	1	1	1	1	1	1	1	1	0.5 ^e^	NA	1	1	1	1	1	1	0	0	0	1	1	1
**Others**																										
26	Gilbert et al., (2000)	48%	0	0.5 ^b^	1	1	1	0	1	0.5 ^c^	NA	1	0.5 ^e^	NA	1	1	NA	NA	NA	0	0	0	0	1	0	0
27	Dion et al., (2010)	63%	1	0.5 ^b^	1	1	1	0	1	0.5 ^c^	NA	1	0.5 ^e^	NA	1	1	NA	NA	NA	0	0	0	1	1	0	1
28	C. R. Rettenmaier et al., (2010)	45%	0.5 ^a^	0.5 ^b^	1	1	1	0	0	0.5 ^c^	NA	1	0.5 ^e^	NA	1	1	NA	NA	NA	0	0	0	0	1	0	0
29	N. Rettenmaier et al., (2010)	50%	0.5 ^a^	0.5 ^b^	1	1	1	0	0	0.5 ^c^	NA	1	0.5 ^e^	NA	1	1	NA	NA	NA	0	0	0	0	1	1	0
30	Imran et al., (2019)	55%	0.5 ^a^	0.5 ^b^	1	1	1	0	1	0.5 ^c^	NA	1	0.5 ^e^	NA	1	1	NA	NA	NA	0	0	0	0	1	1	0
31	Dansk et al., (2016)	90%	1	0.5 ^b^	1	1	1	1	1	1	0.5 ^c^	1	0.5 ^e^	NA	1	1	1	1	1	1	1	1	1	1	1	1
32	Pearce et al., (2016)	98%	1	0.5 ^b^	1	1	1	1	1	1	1	1	1	1	1	1	1	1	1	1	1	1	1	1	1	1
33	Gao et al., (2017)	94%	1	0.5 ^b^	1	1	1	1	1	0.5 ^c^	0.5 ^c^	1	1	1	1	1	1	1	1	1	1	1	1	1	1	1
34	Ehrhardt et al., (2020)	90%	1	0.5 ^b^	1	1	1	0	1	1	1	1	1	1	1	1	1	1	1	1	1	1	0	1	1	1

**NOTE:** 1: criterion met; 0.5: criterion partially met; 0: criterion not met; NA: criterion not applicable; due to their nature, not all CHEERS items were relevant to all studies. ^a^ Did not identify the study as an economic evaluation or use more specific terms. ^b^ Did not provide a perspective or inputs or uncertainty intervals in abstract. ^c^ Did not state why appropriate/why they were chosen. ^d^ Did not apply discount rate but explained reason. ^e^ Did not state why the single study was a sufficient source of clinical effectiveness data. ^f^ Did not give reasons for the specific type of decision analytical model used and did not show the model structure. ^g^ Assumption was not described. ^h^ Non-parametric bootstrapping was used. ^i^ Did not discuss limitations.
